# Acromegaly complications: an update

**DOI:** 10.1210/clinem/dgag114

**Published:** 2026-04-22

**Authors:** Andrea Giustina, Maria Fleseriu

**Affiliations:** Institute of Endocrine and Metabolic Sciences, San Raffaele Vita-Salute University and IRCCS San Raffaele Hospital, Milan 20132, Italy; Pituitary Center, Departments of Medicine and Neurological Surgery, Oregon Health & Science University, Portland, OR 97239, USA

**Keywords:** acromegaly, cardiomyopathy, hypertension, diabetes mellitus, vertebral fractures, cancer

## Abstract

**Context:**

Appropriate diagnosis and treatment of comorbidities is key to managing acromegaly given their adverse clinical impact on quality of life and survival; thus, it is important to increase overall awareness of complications and their individualized management.

**Evidence:**

This review examines the current literature on pathophysiology, diagnosis, and clinical presentation of acromegaly complications, as well as impact of acromegaly therapy and current goals for treatment outcomes of these morbidities.

**Conclusion:**

We focus on the most relevant acromegaly comorbidities including cardiorespiratory, metabolic, bone, and oncologic complications. Selected complications that may determine pharmacologic choices are also evaluated.

Acromegaly is associated with growth hormone (GH) hypersecretion, leading to excess circulating insulin-like growth factor 1 (IGF-I) levels, which exert adverse effects on peripheral organs and physiologic processes (1, 2). Patients commonly experience abnormal growth of bone and soft tissue, dysregulated glucose and lipid metabolism with increased risk for cardiovascular disease (2, 3), and consequent increased mortality risk if not appropriately controlled (4). The treatment goal is to normalize GH and IGF-I levels, as well as signs and symptoms of the disease that often persist despite achievement of biochemical remission (5-8). Early diagnosis and patient-centered comorbidities management favors optimal long-term outcomes (9-13) with superior outcomes reported in Pituitary Tumor Centers of Excellence (PTCOEs) (14, 15).

The purpose of our manuscript is to provide the reader with a comprehensive, updated, and critical review of the available literature including novel research findings on acromegaly comorbidities referring to the modern consensus derived practical guidelines for timing of tests at diagnosis and follow-up (9-12). Management of specific comorbidities is also briefly discussed to complement the available consensus guidelines (9-12). With this aim we performed a comprehensive literature search for English language papers published between September 2015 and September 2025. Search terms included “acromegaly” AND “comorbidities,” “sleep apnea,” “cardiovascular complications,” “bone disease,” “quality of life,” “metabolic complications,” and “cancer,” as well as other terms associated with each respective topic covered. Moreover, terms such as “growth hormone,” “GH,” “insulin-like growth factor 1,” and “IGF-I” were also included because acromegaly signs and symptoms, as well as comorbidities, are mediated either directly by GH or indirectly by elevated IGF-I levels resulting from excess GH action on the liver (16). Non-IGF-I-mediated actions of GH are mainly metabolic effects (eg, lipolysis and increased gluconeogenesis and glycogenolysis, together with decreased tissue glucose uptake) (17) and osteoblast stimulation (18). Circulating IGF-I is the best marker of GH action, and when measured by a validated assay with robust age-related reference ranges, it is the best biochemical marker of disease activity (19). Prevalence and severity of disease complications appear to be related to cumulative exposure to high IGF-I (20).

## Cardiorespiratory complications

**Figure 1:**
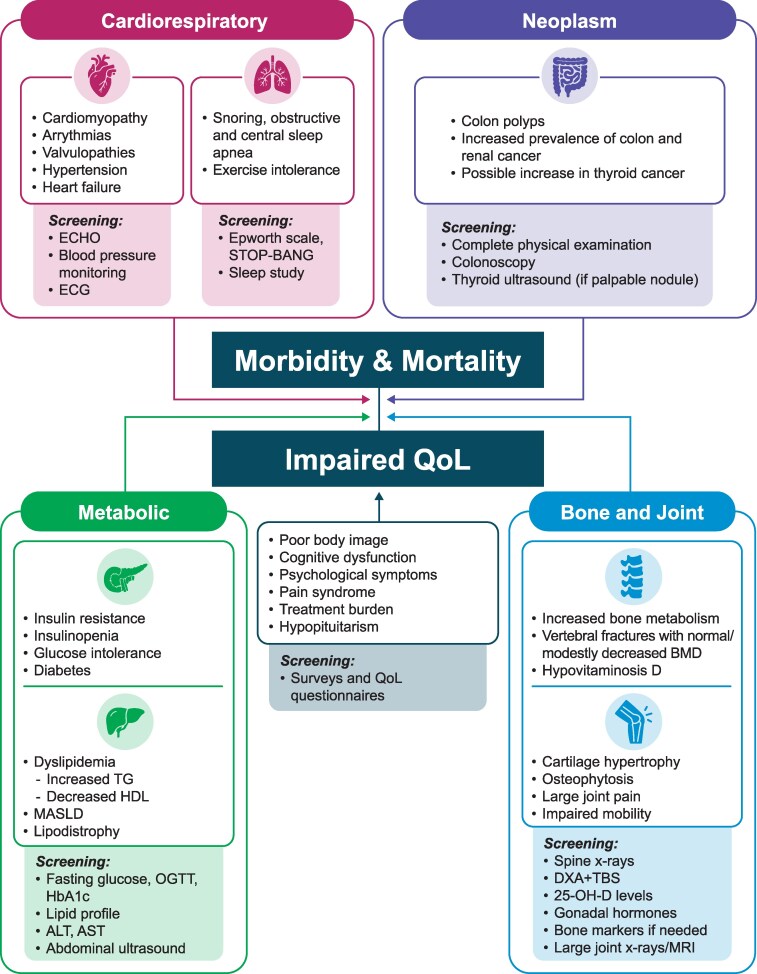
Acromegaly complications. Heart failure is less frequently observed now. Abbreviations: 25-OH-D, 25-hydroxivitamin D; ALT, alanine aminotransferase; AST, aspartate aminotransferase, BMD, bone mineral density; DXA, dual-energy X-ray absorptiometry; ECHO, echocardiogram; ECG, electrocardiogram; HbA1c, hemoglobin A1c; HDL, high-density lipoprotein; MASLD, metabolic dysfunction-associated steatotic liver disease; MRI, magnetic resonance imaging; OGTT, oral glucose tolerance test; QoL, quality of life; STOP-BANG, a screening questionnaire used to assess the risk of obstructive sleep apnea; TBS, trabecular score; TG, triglyceride; VF, vertebral fracture.

Cardiovascular and respiratory comorbidities are frequent clinically relevant findings linked to increased mortality risk (21) (Fig. 1). Cardiomyopathy, arrhythmia, valvular abnormalities, and hypertension occur with varying incidence (22). Sleep apnea and snoring are also common and potentially severe respiratory comorbidities (23). Coexistent cardiac and respiratory complications may lead to decreased exercise tolerance (24).

### Cardiomyopathy

Cardiomyopathy, often an early manifestation of acromegaly, is characterized initially by a reversible hyperkinetic left ventricle. This is followed—especially if acromegaly is not controlled (even without hypertension)—by progressive concentric hypertrophy with a prevalence ranging from 15% to 70% and leading to diastolic dysfunction in 10% to 50% of patients. Combined diastolic and systolic dysfunction is seen mostly in the late stages of acromegaly (25, 26). Speckle-tracking echocardiography and cardiac magnetic resonance (27, 28) showed that treatment partially reversed cardiomyopathy, especially in patients achieving biochemical control. Magnetic resonance imaging is more accurate than echocardiography in detecting ventricular mass, volume, and heart function, and after gadolinium enhancement, it may detect myocardial fibrosis, whereas T1 mapping correlates with myocardial collagen content (28, 29).

IGF-I normalization, through either surgery or medical therapy with somatostatin receptor ligands (SRLs) or pegvisomant, reduces left ventricular mass and improves diastolic function (30-32). Interestingly, SRLs may improve acromegaly cardiomyopathy, including myocardial fibrosis (33), even in some patients who fail to achieve biochemical control (34). Cardiovascular risk in patients undergoing surgery for GH excess is not higher than in patients with nonfunctioning pituitary adenomas (35). Heart failure, although an earlier and severe outcome of acromegaly cardiomyopathy, is fortunately becoming a rare event, occurring in <5% of patients (36), likely as a result of better control of the disease and concomitant risk factors (37).

As per current guidelines, all patients should undergo an electrocardiogram and echocardiography at the time of diagnosis and should undergo periodical long-term echocardiographic follow-up, particularly those who either were not normal before treatment, or did not achieve biochemical control during treatment or have persistent arterial hypertension (11).

### Arrhythmias

Up to 40% of patients with acromegaly may be diagnosed with arrhythmias, including atrial fibrillation with cardiomyopathy (26-39). Patients also may present with prolonged QT interval (40), elevated beat-to-beat QT interval variability, and a higher prevalence of late potentials (41). Diagnosis of prolonged QT interval is relevant prior to treatment, in particular for pasireotide, which may be associated with QT interval prolongation (42).

### Valvular abnormalities

Frequent echocardiographic findings (43, 44) include tricuspid regurgitation, although mitral and aortic valves also may be involved (45). Despite the achievement of biochemical control, valvular disorders are rarely reversible (26). Cabergoline, even at low doses, should be used with caution in patients with acromegaly and severe heart valve regurgitation (46).

### Hypertension

Hypertension has a variable prevalence, ranging from 30% to 60% of patients with acromegaly in different series, particularly in those with active disease (47). Furthermore, hypertension in acromegaly is more frequent and may occur earlier than in individuals without acromegaly, matched for sex, age, body mass index (BMI), and smoking habits, involving predominantly, although not exclusively diastolic blood pressure. Importantly, hypertension prevalence does not differ by sex and is unfrequently related to family history of hypertension (48). Hypertension increases mortality risk significantly, especially when associated with cardiomyopathy or other cardiovascular risk factors (49, 50).

Persistent hypertension, even after controlling acromegaly, is not uncommon (51) and may be due, particularly in older patients, to the coexistence of primary hypertension as well as partial reversibility of volume expansion, peripheral vascular resistance, and sleep apnea (1).

Although not used routinely, 24-hour ambulatory blood pressure monitoring has been recommended (52), as it is more sensitive and correlates better with ventricular mass than office blood pressure measurement. This can also have higher accuracy for assessing the efficacy of antihypertensive treatment. Nondipper behavior was reported to be significantly associated with the hormonal profile (53).

Surgery improves blood pressure levels, at least in the short-term, with restored day/night rhythm, particularly in individuals with controlled disease (54). The impact of SRLs on blood pressure is controversial, being reported either to improve or to have no significant effect. SRLs may have a beneficial antihypertensive effect due to biochemical control and are less likely to have a pleiotropic effect (55). The observation that pegvisomant may improve hypertension (56) also confirms the existence of a tight link between hormonal excess and increased blood pressure levels. Controlled studies evaluating or comparing different antihypertensive treatments in acromegaly are not available. Therefore, any recommendations for specific antihypertensive treatment in acromegaly have been so far issued on consensus guidelines, which suggest following general population guidelines (11). However, since sodium overload may occur, an epithelial sodium channel blocker (eg, amiloride) may be clinically helpful (57).

### Sleep apnea

Sleep apnea is highly prevalent and present in up to 80% of patients at diagnosis (23). Upper airway obstruction due to soft tissue swelling is the main pathophysiological feature (58) even if central sleep apnea is present in about 30% of patients. Obstructive sleep apnea is associated with cardiovascular complications (59, 60) and with disordered sleep and heavy snoring, which may lead to impaired quality of life (QoL), and mood and cognitive symptoms (61). Sleep apnea is one of the most underdiagnosed though clinically impactful complications (62), due in part to reduced access to sleep units (63). Moreover, assessment of sleep apnea, recommended in all patients with acromegaly at the time of diagnosis (11), may require polysomnography, which is not a routinely performed test in all countries. Administration of sleep questionnaires, such as the Epworth Sleepiness Scale, may be a viable alternative but is time consuming (64), and follow-up of changes in polysomnographic sleep apnea scores is logistically challenging. Interestingly, the availability of new technologies and artificial intelligence may transform the approach to the diagnosis of sleep apnea in acromegaly (65, 66).

Treatment of acromegaly may improve obstructive sleep apnea by reducing pharyngeal swelling (67-69). However, even if biochemical control of acromegaly is achieved, sleep apnea does not improve in all patients. Few studies with SRL treatment have shown improvement (70) in sleep apnea, yet SRLs have, nevertheless, been considered as presurgical treatment for facilitating intubation (71), though not proven to improve outcomes. Masks for continuous positive airway pressure may be necessary in some patients with the most severe forms (11).

Inclusion of a sleep specialist in a PTCOE in the future might partially alleviate the difficulties in diagnosis and treatment for sleep disorders in patients with acromegaly.

### Cardiopulmonary response to exercise

Impaired cardiopulmonary function may reduce exercise capacity to only 60% to 85% of normal sex- and age-adjusted values (72). Impaired physical performance may appear early in the disease course, primarily due to diastolic dysfunction despite a resting normal systolic function (24). Decreased ejection fraction at peak exercise inversely correlates with age and disease duration (73).

The main feature of impaired cardiopulmonary response to exercise is reduced maximal oxygen consumption (74). Increased lung volume and distensibility with decreased elasticity (75), together with altered rib cage geometry, may reduce ventilatory efficiency with inadequate ventilatory response to exercise demand, resulting in low workload capacity (76). Finally, the recently recognized myopathy in acromegaly, characterized by ectopic lipid deposition and decreased muscle performance, also may contribute to impaired exercise capacity (77, 78).

GH and IGF-I may acutely impact cardiorespiratory function (24); octreotide may have beneficial effects on exercise tolerance (79) and improve ejection fraction (80), although increased maximal oxygen consumption is limited (81). Specific measures to improve exercise capacity include short-term regular exercise programs that may improve cardiopulmonary function and exercise time and capacity (82), even in patients with controlled disease.

## Metabolic complications

### Glucose metabolism

Glucose metabolism is frequently altered in acromegaly, with approximatively one-third of patients already living with diabetes mellitus at diagnosis (83, 84). GH impairs glucose tolerance by decreasing peripheral sensitivity to insulin and glucose uptake and increasing hepatic gluconeogenesis (85, 86). Diabetes mellitus has a significant clinical impact in patients with acromegaly, increasing the risk of morbidity and mortality by up to 60% (87).

Glucose homeostasis should be evaluated at diagnosis by assessing fasting glucose and glycated hemoglobin levels (11). Measuring the blood glucose level at 120 minutes of the oral glucose tolerance test (OGTT) may better define the patient's glycemic status. OGTT, used in selected instances to confirm acromegaly diagnosis, is relatively contraindicated in patients with diabetes and/or those receiving antidiabetic treatment (88).

In fact, acromegaly control is crucial to the management of hyperglycemia, as β-cell function was normalized in ∼20% of patients after successful pituitary surgery if there was normal anterior pituitary function (89, 90). In contrast, due to their direct negative impact on insulin secretion, SRLs may adversely impact glucose metabolism. However, octreotide, lanreotide, and paltusotine (8) have marginal, if any, hyperglycemic actions (91, 92). On the other hand, pasireotide binds not only to somatostatin 2 receptor (SST2) but also to SST5, which frequently disrupts glucose metabolism by reducing insulin secretion and glucagon and incretin effects, particularly in patients with preexisting hyperglycemia (93, 94), and is not recommended in patients with poorly controlled diabetes (7, 95). Therefore, fasting and postprandial glucose should be monitored, particularly in patients taking pasireotide, because hyperlipidemia, hypertension, and older age at diagnosis predispose patients to a higher risk of hyperglycemia (85). Hyperglycemia can occur rapidly after starting treatment and can be effectively managed with incretin therapy or insulin if needed (96-98). Conversely, pegvisomant may enhance glucose metabolism in certain patients by mitigating insulin resistance, partly through mechanisms independent of biochemical control. It should be considered as monotherapy or in combination with SRLs for individuals with impaired glucose metabolism (99, 100), and should be considered either as monotherapy or with SRLs in patients with impaired glucose metabolism (7).

Choice of antidiabetic medications is guided by severity of hyperglycemia, undesired effects and patient preference, and cardiovascular and kidney comorbidities. In fact, structural and functional renal alterations found in active acromegaly, such as renal hypertrophy, high glomerular filtration rate, nephrolithiasis due to hypercalciuria, and microalbuminuria, may be only partly reversible after GH and IGF-I normalization (101-103). Diabetes and hypertension contribute to an increased risk of renal insufficiency (104).

Initially, nonpharmacologic measures and metformin, unless contraindicated, can be used. Sodium–glucose cotransporter 2 inhibitors and glucagon-like peptide-1 receptor agonists may have cardiovascular and renal protective effects. However, sodium–glucose cotransporter 2 inhibitors, which may have diuretic effects, should be used with caution in patients with suppressed insulin who are taking pasireotide due to the risk of diabetic ketoacidosis (105).

### Lipid metabolism

Atherogenic dyslipidemia patterns occur in about one-third of patients with acromegaly (106), with coexistent hyperglycemia and/or metabolic syndrome. The typical lipid profile is characterized by hypertriglyceridemia with reduced high-density lipoprotein cholesterol and increased lipoprotein(a) levels (106). GH excess may enhance lipolysis with consequent increase of circulating free fatty acids, which may in turn increase insulin resistance (107).

Treatment of acromegaly may lower serum triglycerides and lipoprotein(a) and increase high-density lipoprotein cholesterol (108). Lipid-lowering treatments should be used according to guidelines for the general population (109).

Increased triglycerides with dyslipidemia may be associated with metabolic dysfunction-associated steatotic liver disease (MASLD) and hepatic fibrosis (110, 111). Interestingly, MASLD may not be related to acromegaly disease control (110); on the contrary, it may be even more frequent in patients with GH deficiency after acromegaly treatment (112-114).

Since patients with acromegaly undergo at least 1 abdominal ultrasound in some countries (particularly before starting SRLs), a preliminary echographic liver evaluation, as well as serum transaminase measurements, could be beneficial at diagnosis because MASLD may be associated with increased cardiovascular risk. Further specific hepatologic assessments should be performed according to guidelines for the general population. In patients with clinically relevant MASLD, caution is necessary when combining SRLs with pegvisomant, which has an increased risk of liver enzyme abnormalities (7).

Acromegaly is associated with a specific lipodystrophic pattern as evidenced when body composition is evaluated with dual-energy X-ray absorptiometry (DXA), which importantly cannot be captured by simple BMI calculation (115). It is characterized by reduced visceral and subcutaneous adipose tissue, with ectopic lipid and adipose tissue deposition in the muscles (116). Increased lean body mass may be due more to expansion of extracellular water rather than increased skeletal muscle which, in fact, is characterized by decreased performance, particularly in uncontrolled disease (117, 118). Because DXA is used in all patients to assess bone mineral density (BMD) and morphometric vertebral fractures (VFs), we suggest that body composition be evaluated at the same time when possible.

Paradoxically, surgery or medical GH-lowering treatments may increase central adiposity with either decreased or unchanged lean body mass likely due to subsequent GH deficiency (119).

## Osteopathy

GH is osteoanabolic (120-122), but hyperactivity of the GH/IGF-I axis causes excess skeletal turnover, assessed by specific biochemical markers (121, 122); trabecular bone microarchitecture is mostly damaged (122-124). This, in turn, increases skeletal fragility, associated with a high prevalence and incidence of sex-independent morphometric VF risk already at diagnosis (125). In fact, seminal studies published 20 years ago (126, 127) have been confirmed worldwide, identifying skeletal fragility as an emerging major complication of acromegaly (128-130) (Fig. 1). VFs can be predicted based on disease activity, as expressed by random circulating GH levels (especially if >12 ng/mL) (131) and disease duration due to long diagnostic delay (132). In fact, biochemical remission of the disease decreases fracture risk (133); however, it may persist in patients with controlled acromegaly and prior VFs, hypogonadism, vitamin D deficiency, coexisting diabetes mellitus, and hypopituitarism, the latter particularly if glucocorticoid replacement has been supraphysiological (134-137).

VFs are a common and early event in the natural history of acromegaly affecting around one-third of patients (131) and one of the most important determinants of refracture risk, mortality, and QOL (138), vertebral morphometry using spine X-rays, DXA, or even, opportunistically, thoracic X-ray performed for anesthesiology or other diagnostic purposes is necessary (131). In fact, VFs also may occur in patients with apparently normal or slightly reduced lumbar spine BMD measured by DXA (126, 139) since deteriorated bone quality is the main feature of acromegaly osteopathy (122, 140). Measures of vertebral bone quality such as trabecular bone score can be provided by DXA and may have a practical role in the assessment of skeletal status (141), especially in PTCOEs where a bone expert is available (142). When used in research, high-resolution peripheral quantitative or cone beam computed tomography have shown increased cortical porosity and reduced cortical strength in patients with acromegaly and VFs vs those without VFs (140, 143).

Monitoring bone status during follow-up depends on the risk of fracture assessed at diagnosis and the degree of biochemical control after acromegaly treatment, as well as coexisting fracture risk factors (139). If BMD is low at diagnosis, measurement of femoral neck BMD using DXA every 12 to 18 months may be useful because it can predict incident VF risk (139). DXA-based vertebral morphometry and bone quality assessments should be repeated at follow-up, particularly in patients with prevalent VFs, decreased BMD, and uncontrolled acromegaly (144).

Importantly, the frequent presence VFs may impact the choice of GH-lowering treatments, which may have a differential effect on progression (135) or occurrence of new incident VFs in high-risk patients (139). Biochemical control is associated with a decreased risk of fractures (145). However, in a single study, patients with uncontrolled acromegaly who were taking pasireotide but not pegvisomant possibly experienced a protective effect against incident VFs (146), suggesting that treatment with pasireotide may be preferable in patients with resistant disease (144) and prevalent VFs (138).

Hypovitaminosis D is omnipresent in the general population (147) and in patients with acromegaly and high IGF-I (148), who have reduced free vitamin D levels due to increased vitamin D binding protein (149). Low vitamin D levels may increase the risk of incident VFs and in acromegaly, cholecalciferol supplementation may be associated with reduced incidence of VFs (150). Antiresorptive therapies may be preferable first-line treatments (as in the general population), based on high bone turnover, which characterizes acromegaly osteopathy (133).

Balance abnormalities also are common in patients with long-term acromegaly and can increase risk of falls with fractures in patients at higher risk of severe osteopathy (151).

Integration of dentistry into interdisciplinary care is also desirable and collaboration models reduce delays in diagnosis of dental manifestations such as dental diastema, mandibular overgrowth and prognathism, overbite and malocclusion (1), while also improving the management of craniofacial complications encountered in some patients (152, 153).

## Arthropathy

The presence of arthropathy in acromegaly is increased 4- to 12-fold vs the general population, with joint cartilage changes due to increased GH and IGF-I levels (138, 154), which stimulate chondrocyte synthesis and replication, as well as the production of glycosaminoglycans and proteoglycans that alter cartilage structure (155). In the initial stages, cartilage and periarticular tissue hypertrophy (156) are potentially reversible, especially if biochemical control is achieved, underscoring the importance of early diagnosis and treatment for this complication (156-158). If GH hypersecretion persists, a degenerative process develops, with appearance of cysts, osteophytes, and excessive growth and thickness of cartilage tissue, with unique radiologic findings (159). At this point, the joint is irreversibly and progressively damaged, with narrowed joint space even if the disease is well controlled (159), and becomes severely symptomatic (156, 159). This clinical picture is not uncommon, affecting 10% to 15% of patients, particularly women and older patients with uncontrolled GH/IGF-I levels (160). Pain and loss of function characterize arthropathy, leading to deterioration of QoL, disability, and depression (161).

Although early joint alterations may be reversible after SRL treatment (20), late stage arthropathy does not improve after long-term medical treatment and management in patients with acromegaly should be similar to that of the general population (156). Interestingly, a relatively short-term, tailored physical activity program is beneficial even in patients with controlled acromegaly (162). Since advanced stages of disease are characterized by progressive disability, joint replacement is often necessary and the risk of requiring this procedure on the shoulder, knee, or hip is more than double that of the general population (153). Notably, patients with acromegaly had a 3.5-fold increased risk of repeat hip surgery.

Carpal tunnel syndrome, a frequent and early complication (163), often leads to consequent neuropathic manifestations (164), median nerve enlargement, and edema (165, 166). A retrospective cohort study of >500 patients with acromegaly in Sweden reported a 6-fold higher incidence of surgery for carpal tunnel syndrome vs the general population, particularly in females and primarily before an acromegaly diagnosis (167).

## Neoplasms

GH and IGF-I may be mechanistically involved in cancer development and progression (168). Most studies, although not all, have reported a possible increased risk of malignancies, especially colon cancer, in acromegaly (169). Importantly, high prevalence of colon polyps, a known precancerous condition, has been consistently reported (170) (Fig. 1). However, while meta-analyses had high heterogeneity (171-173), 3 large, longitudinal studies published in 2025 better defined cancer risk in acromegaly (174-176).

In a longitudinal Korean cohort study of 2382 patients with acromegaly and 11 910 controls with adjustments for age, sex, and metabolic factors, the risk of overall cancer in patients with acromegaly (10.2%) was double that of controls (5.9%) (hazard ratio [HR], 1.90 [95% CI, 1.63-2.22]). Brain cancer had the highest relative risk (HR, 6.80 [2.83-16.38]), followed by lymphoma, multiple myeloma, thyroid, pancreatic, and colorectal cancers (174).

In a prospective US longitudinal study on 598 patients with acromegaly, cancer prevalence was 22.6%, almost double that of patients with nonfunctioning adenomas from the same referral population (12.7%) (odds ratio [OR], 1.99 [95% CI, 1.34-2.97]). Overall, cancer standardized incidence ratio of 1.78 (95% CI, 1.51-1.81) was almost superimposable to that of the Korean study; furthermore, it was related to a cumulative exposure to IGF-I (175).

A similar prevalence of solid cancers (21.3%) was reported in a cohort of 470 patients with acromegaly in Israel with a 10-year follow-up (176). They also had significantly higher risk vs controls for thyroid cancer (OR, 5.1 [95% CI, 2.3-11.0]), but the incidence of colorectal, prostate, and renal cancers was not increased (176). However, another cohort study reported increased renal cancer risk (177). Smoking and age >50 years were related to a higher risk of premalignant/colon cancer lesions (178). While large studies often report increased cancer incidence, increases in malignancy-associated mortality have not been consistently reported (21). Uncertainty about the real clinical impact of cancer risk in acromegaly also is reflected in the lack of specific guidelines for cancer screening (179). At this time, recommendations for screening patients with acromegaly follow those for the general population, even for colon cancer, despite the higher prevalence of colon polyps in these patients (180, 181). Colonoscopy is recommended at the time of acromegaly diagnosis, but practices differ between countries.

## Quality of life

Despite efforts to increase awareness, patients with acromegaly still experience a prolonged diagnostic delay, at times >10 years (181). This leads to prolonged cumulative exposure to high levels of GH and IGF-I. In turn, this further adds to the disease burden due to the potentially irreversible complications that impact QoL (182). A recent meta-analysis (8 studies, 1387 patients) showed an increased risk of depression (risk ratio [RR], 1.8 [95% CI, 1.3-2.50]) and anxiety (RR, 1.9 [95% CI, 1.1-3.2]) vs patients with nonfunctioning pituitary adenomas (183).

Impaired QoL based on disease-specific questionnaires such as the Acromegaly Quality of Life questionnaire (AcroQoL) (184) shows little correlation with biochemical outcomes in ≥33% of patients. However, effective therapy improves AcroQoL scores after all treatment types (185), especially surgical adenoma resection. Despite controlling many clinical disease features, frequent SRL injections may reduce patients’ sense of well-being, even in those considered biochemically controlled (186). Frequent gastrointestinal and local injection site side effects may increase treatment burden (187). Interestingly, oral octreotide (188-190) and paltusotine (191, 192) could decrease treatment burden and improve QoL (193). Adding pegvisomant to long-acting SRLs improves the AcroQoL physical domain independent of biochemical control (194). In addition to arthropathic, muscular, and psychological complications (193), QoL may be adversely impacted by age of disease onset, female sex, obesity, duration of disease, fatigue, hypopituitarism, and conventional radiotherapy (195-197).

Poor correlation between patient- and physician-reported outcomes reinforces a communication gap (198). Tools used by clinicians for improving diagnosis and follow-up such as SAGIT, which was the first evaluation to include symptoms and complications for a holistic physician evaluation (199), should be integrated with patient-reported outcome measures to include patients’ perception of the journey and disease/treatment burden with shared decision-making (200).

## Mortality

Acromegaly continues to carry an increased risk of mortality, as reported by several recent studies from parts of the world (201-203), although this risk is now lower than in earlier reports (204, 205). Diagnostic delay has been consistently shown to be a key predictor of mortality risk (181, 203). Disease control, essential to slow progression, if not reversal of complications, is crucial to reducing adverse mortality rates (206). Interestingly, in a recent retrospective analysis of >2000 patients from the ACROSTUDY who received pegvisomant and were followed for a median of 4 years, mortality due to cardiovascular, cerebrovascular, cancer, and respiratory causes significantly increased when associated with higher IGF-I levels, with ORs ranging from 1.57 to 1.97 (207). These results underscore that despite advances in treatment (208), patients who do not achieve early disease control have an adverse long-term prognosis. Intriguingly, a recent survey of several centers of excellence around the world providing multidisciplinary care (209) reported that with any medical treatment option, a relevant number of patients with acromegaly still remained uncontrolled (15). In addition to diagnostic delay, therapeutic inertia seems to be a pillar of poor prognosis (210). In fact, shortening the time to switch to another treatment (7) or simply to optimize dose uptitration of the same treatment (211-213) in patients with uncontrolled acromegaly (214) should be prioritized, because maintaining patients on an ineffective therapy increases the cumulative exposure to high GH and IGF-I levels.

## Conclusion

Acromegaly is a chronic disease still highly burdened by severe cardiorespiratory, metabolic, and oncologic complications that have a significant impact on QOL and lifespan. Since delays in diagnosis are still very long, a thorough assessment of complications present at the time of diagnosis is crucial because the patient may already have been exposed to high GH and IGF-I levels for several years. In addition to reducing diagnostic delay—a modifiable risk factor—rapid and effective biochemical disease control and appropriate treatment of already existing complications should be prioritized to optimize QOL and prognosis. A multimodal management provided by PTCOEs may provide optimal surgical and medical management to counteract therapeutic inertia, as well as enable specialist consultations for follow-up and treatment of systemic complications. These approaches will ideally improve prognosis, and ultimately mortality rates, particularly for truly resistant forms of acromegaly.

## Data Availability

Data sharing is not applicable to this article, as no datasets were generated or analyzed during the current study.

## Abbreviations

AcroQoL: acromegaly quality of life questionnaire
BMD: bone mineral density
DXA: dual-energy X-ray absorptiometry
GH: growth hormone
HR: hazard ratio
IGF-I: insulin-like growth factor 1
MASLD: metabolic dysfunction-associated steatotic liver disease
OGTT: oral glucose tolerance test
OR: odds ratio
PTCOE: Pituitary Tumor Center of Excellence
QoL: quality of life
RR: risk ratio
SAGIT: “signs & symptoms, associated comorbidities, GH levels, IGF-I levels, and tumor profile”
SRL: somatostatin receptor ligand
SST: somatostatin receptor type
VF: vertebral fracture
